# Exploring existing malaria services and the feasibility of implementing community engagement approaches amongst conflict-affected communities in Cameroon: a qualitative study

**DOI:** 10.1186/s12936-024-04934-x

**Published:** 2024-05-20

**Authors:** Margaret Ebob Besem E.O, Elisabeth G. Chestnutt, Laura Donovan, Ann-Sophie Stratil, Helen Counihan, Claude Ngwayu Nkfusai, Helen Hawkings, Blanka Homolova, Kolawole Maxwell, Kevin Baker, Yakouba Zoungrana, Elvis Asangbeng Tanue, Glennise Ayuk, Noukeme Bibiche Modjenpa, Alain Metuge, Isabelle Nganmou, Dorothy Achu, Samuel Wanji, Elizabeth Berryman, Lundi-Anne Omam

**Affiliations:** 1 Malaria Consortium, The Green House, 244-254 Cambridge Heath Rd, London, E2 9DA UK; 2 Department of Public Health and Hygiene, Faculty of Health Sciences, University of Buea, P.O Box 63, Buea, Cameroon; 3 Department of Public Health, School of Nursing and Public Health, University of Kwa-Zulu Natal, Durban, South Africa; 4 Malaria Consortium Nigeria, No 33 Pope John Paul Street, Off Gana Street, Maitama, Abuja, FCT Nigeria; 5 Karolinksa Institute, Nobels Väg 15 A, 171 77 Stockholm, Sweden; 6 Reach Out Cameroon (REO), P.O Box 88, Buea, Cameroon; 7 Konmofamba Actions Sans Frontieres (KASAFRO), Penja, Cameroon; 8 National Malaria Control Programme, Ministry of Public Health, Yaoundé, Cameroon; 9 Department for Microbiology and Parasitology at the University of Buea, Buea, Cameroon; 10 Research Foundation in Tropical Diseases and Environment, Buea, Cameroon; 11 Department of Public Health and Primary Care, University of Cambridge, Cambridge, UK

**Keywords:** Malaria services, Community engagement, Conflict-affected communities, Cameroon

## Abstract

**Background:**

Cameroon is one of the countries with the highest burden of malaria. Since 2018, there has been an ongoing conflict in the country, which has reduced access to healthcare for populations in affected regions, and little is known about the impact on access to malaria services. The objective of this study was to understand the current situation regarding access to malaria services in Cameroon to inform the design of interventions to remove barriers and encourage the use of available services.

**Methods:**

A qualitative research study was carried out to understand the barriers preventing communities accessing care, the uptake of community health worker (CHW) services, and to gather perceptions on community engagement approaches, to assess whether these could be an appropriate mechanism to encourage uptake of community health worker (CHW) services. Twenty-nine focus group discussions and 11 in-depth interviews were carried out between May and July 2021 in two regions of Cameroon, Southwest and Littoral. Focus group discussions were held with CHWs and community members and semi-structured, in-depth interviews were conducted with key stakeholders including regional government staff, council staff, community leaders and community-based organisations. The data were analysed thematically; open, descriptive coding was combined with exploration of pre-determined investigative areas.

**Results:**

The study confirmed that access to healthcare has become increasingly challenging in conflict-affected areas. Although the Ministry of Health are providing CHWs to improve access, several barriers remain that limit uptake of these services including awareness, availability, cost, trust in competency, and supply of testing and treatment. This study found that communities were supportive of community engagement approaches, particularly the community dialogue approach.

**Conclusion:**

Communities in conflict-affected regions of Cameroon continue to have limited access to healthcare services, in part due to poor use of CHW services provided. Community engagement approaches can be an effective way to improve the awareness and use of CHWs. However, these approaches alone will not be sufficient to resolve all the challenges faced by conflict-affected communities when accessing health and malaria services. Additional interventions are needed to increase the availability of CHWs, improve the supply of diagnostic tests and treatments and to reduce the cost of treatment for all.

**Supplementary Information:**

The online version contains supplementary material available at 10.1186/s12936-024-04934-x.

## Background

Cameroon is among the 29 countries that account for 96% of malaria infections globally [1]. Between 2011 and 2018, progress was made to reduce malaria prevalence and increase household ownership of insecticide-treated nets [2]. However, in 2019, malaria was still the cause of over 40% of all deaths in Cameroon [3]. Since 2018, there has been an ongoing armed conflict in the Northwest (NW) and Southwest (SW) regions of Cameroon, known as the Anglophone crisis. According to The United Nations Office for the Coordination of Humanitarian Affairs (UNOCHA), as of August 2021, the crisis is estimated to have displaced over one million people, of which approximately 200,000 have fled to the Littoral region [4, 5].

The conflict has impacted access to healthcare services, with reports suggesting 29% of health centres in these regions are no longer functioning [6]. Many citizens are also too scared to visit the remaining facilities due to safety concerns or are unable to afford treatment due to loss of livelihoods [7]. This situation is increasingly problematic for internally displaced people (IDPs) who are also at higher risk of contracting malaria, due to less secure housing and reduced access to malaria prevention methods and treatment services. In addition, IDPs that have relocated to the Littoral region have moved to an area where the malaria prevalence is higher than their home region [8].

Health data is also scarce, primarily due to the closure or destruction of health facilities. The most recent demographic and health survey reported malaria prevalence in the SW region averaged 10%, however these data were only collected from urban areas and are not representative of the entire region [8]. Studies conducted prior to the conflict reported a malaria prevalence varying from 9 to 56%, depending on the season and location, and district medical officers have reported a general increase in the number of malaria cases reported [7, 9].

To improve access to malaria prevention and treatment services the Ministry of Health in Cameroon has been implementing a Community Health Strategy [10]. This strategy aims to ensure that by 2025, 60% of populations living more than 5 kms from a health facility have access to high-quality healthcare services through multi-purpose community health workers (CHWs) [10]. The strategy also aims to increase demand for community health services through communication and community dialogue structures [10]. The success of this strategy relies in part on the degree of CHW acceptability at the community level [11].

This qualitative study was conducted as part of a larger research study—alongside a quantitative component—to understand the challenges IDPs face when accessing malaria services, factors that influence their use of CHWs, and assess the preference of key stakeholders for different community engagement interventions to improve uptake of malaria prevention measures and services.

## Methods

### Study design

The research employed a qualitative approach, comprising of focus group discussions (FGDs) with community members and CHWs and in-depth interviews (IDIs) with key stakeholders, to understand the challenges and subsequently design context-appropriate interventions to improve access and use of available health services. FGDs covered topics such as knowledge of malaria, access to healthcare services, perception and use of CHW services, and knowledge and perception of community engagement approaches. IDIs discussed the barriers communities face accessing treatment, community perceptions and use of CHW services, and feasibility of implementing community engagement approaches.

### Sampling methods and study area

**Fig. 1:**
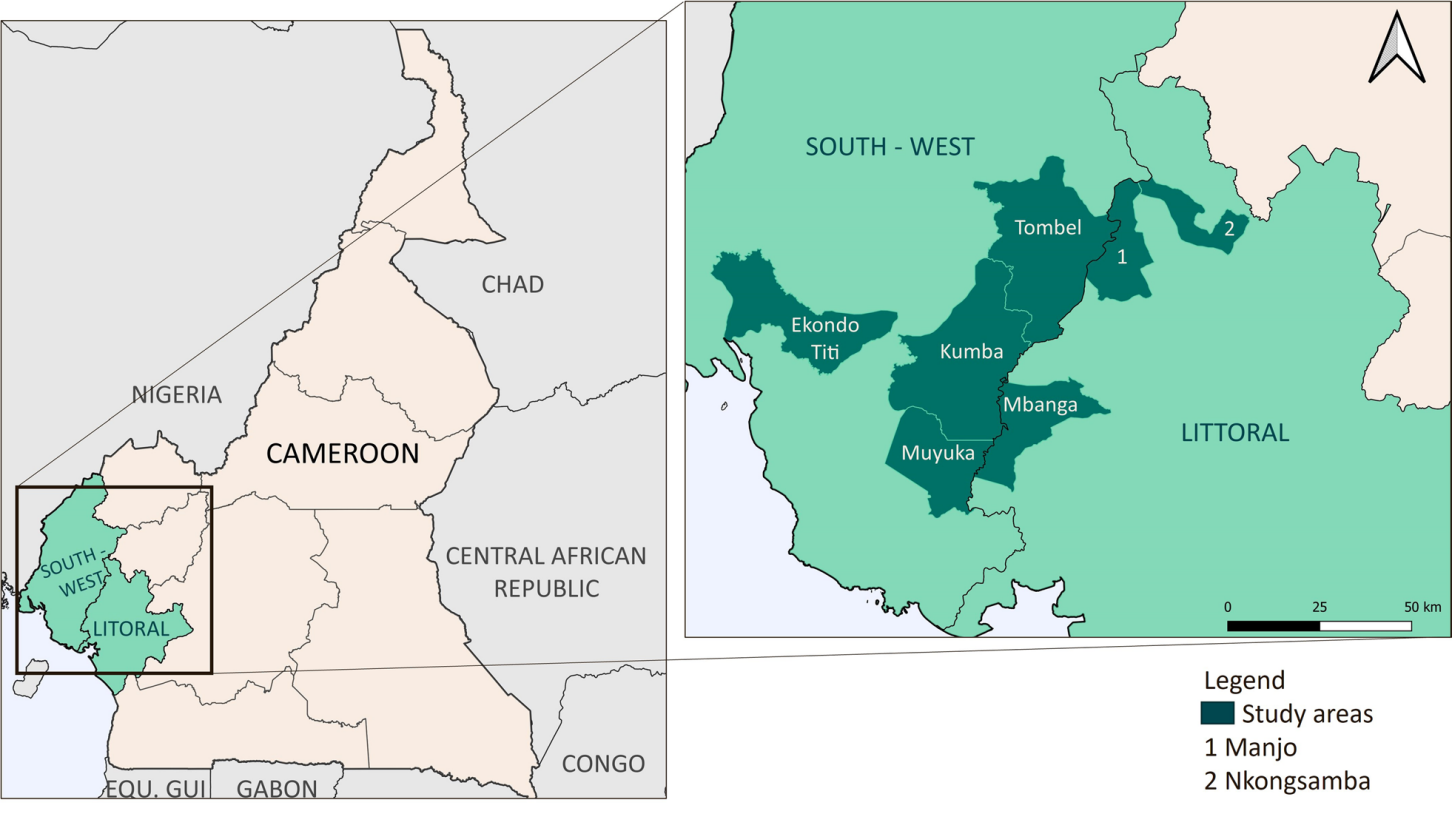
Cameroon map with indicated study areas

This study was conducted between May and July 2021 in two regions of Cameroon—the conflict-affected SW region and Littoral region which hosts a high number of IDPs (Fig. 1).

The study was conducted in four health areas of the SW region – Ekondo-Titi, Kumba, Muyuka and Tombel. These health areas were selected as conflict-affected study sites as there was a presence of armed groups, frequent active fighting and kidnapping, public service disruption and damage and frequent government lockdowns [12]. These sites also had a paucity of data on service availability due to the conflict [13]. Three health areas in the Littoral region were also included—Manjo, Mbanga, and Nkongsamba—as they host a high number of IDPs.

Communities chosen for the study were selected at random, through convenience sampling and in consultation with local leaders and CHWs, to ensure a relevant IDP population was available for FGDs.

### Data collection

#### Focus group discussions

Two FGDs were carried out with community members at each study site, one with men and one with women. The selection criteria for these FGDs were to be resident in the study area, to be over 18, and to be either a man or woman with a key role in household treatment-seeking or health management. Each FGD had 8–12 participants and were led by a trained male or female facilitator and a male or female research assistant, depending on whether the focus group was with female or male participants. The research teams were supervised by the female project operational research specialist.

The criteria for selection were explained by the research team to health facility staff, district councillors and community leaders who then selected the participants. Participants were not known to the researchers and interviewers prior to the study. The FGDs took place in venues selected by counsellors and community leaders. All venues were community halls or dedicated community centres for community activities. All IDIs took place in the interviewees place of work. The community halls, workplaces and timing of interviews ensure privacy. Each interview and FGD took an average of one hour. All interviews were face to face.

The facilitator and assistant were provided with detailed interview and FGD topic guides. The FGD topic guides (Additional file 1) covered access to healthcare services and in particular malaria services, treatment-seeking practices, CHW utilization and the perceived quality of CHW services, and knowledge and perceptions of three community-based interventions—the community dialogue approach, village health committees and community scorecards.

One additional FGD was carried out with CHWs in each of the study sites (four in the SW and three from the Littoral region). Each FGD included approximately 16 CHWs and participants and moderators were mixed gender. The selection criteria were to have been a CHW providing malaria case management services in the community for at least two years. The topic guides covered whether malaria is a problem, what services CHWs provide, challenges faced when providing services and knowledge and perceptions of the three community-based interventions proposed to communities.

The study was explained, and consent requested from all participants in the FGDs. Participants were excluded if they were under the age of 18, unable to give informed consent or communication was limited. Sessions were conducted in the local language (Pidgin English), audio-recorded, and later transcribed and translated to English by field supervisors of the respective project sites.

#### In-depth interviews

IDIs were conducted by trained project field supervisors who used interview guides. Participants included: community leaders, city council and regional government staff, Ministry of Health officials, National Malaria Control Programme staff, local government officials and one community-based organisation. All interviewees were male, and interviewer were mixed genders (two female and three male). All participants gave informed consent prior to the interviews.

The topics covered in the interviews were adapted to the respondents’ roles (Additional file 2). The IDIs with community leaders and community-based organisations discussed barriers in access to treatment, perceptions and use of CHW services, and feasibility of implementing three community-based interventions in their communities. The IDIs with city, regional and national government staff focussed on malaria services provided by CHWs, barriers to accessing healthcare in conflict-affected communities and their perceptions and feasibility of community-based interventions to address these barriers. Interviews were conducted in English, including those with community leaders. All interviews were audio-recorded and transcribed by hand by the project field supervisors.

A total of 29 FGDs were conducted, 22 in the SW region and seven in the Littoral region. Community members and CHWs participated in 22 and seven FGDs, respectively. A total of 11 IDIs were carried out, seven in the SW region and four in the Littoral region (Table 1).

**Table 1:** Number of FGDs per project site, by type of study participant

Region	FGDs		IDIs			
Health area	Community members	CHWs	Community leaders	Community-based organisations	Council staff	Regional government staff
Littoral						
Manjo	2	2	2	–	1	1
Mbanga	–	1	–	–	–	–
Nkongsamba	2	–	–	–	–	–
Southwest						
Ekondo-Titi	4	–	2	–	–	–
Kumba	6	2	–	1	1	–
Muyuka	4	2	1	–	–	–
Tombel	4	–	1	–	–	1
Total	22	7	6	1	2	2

#### Data analysis

Data from FGDs and IDIs were analysed thematically; open, descriptive coding was combined with exploration of pre-determined investigative areas. The analysis was conducted using the *Atlas Ti* version 9.0 software.

## Results

### Access to care

Community members reported that accessing healthcare services has become more difficult since the conflict started. In the SW region, community members reported physical barriers to accessing care, mainly due to closure and destruction of hospitals or health facilities, a lack of transport links (affected by rainfall), and restrictions on movement due to fear of insecurity.

“*Distance and transport to the health centre. With the water we do not have the transport to reach the hospital*” (FGD with community members, Ekondo-Titi, SW).

Respondents in this region also reported a lack of trust in the government, this prevented them from seeking treatment at services provided by the government. In addition, respondents reported a perception that the doctors at the remaining health facilities were poor quality. In the Littoral region, respondents said the key challenges limiting access to health services were the influx of IDPs which had increased the number of people using healthcare services, but no additional resources had been provided to manage the increase in use. In both regions, respondents reported that the presence of CHWs had improved access to care as they reduced the distance to, and cost of services.

*“It has affected in the sense that there are no good doctors to take care of the population when they are sick and the crisis has made everything worse”* (FGD with community member, Muyuka, SW)

### Factors influencing the use of CHWs

#### Availability

Several respondents in the SW region reported that they visit CHWs because there is an absence of hospitals and health facilities, and because CHWs are available close to where they live.

*“Hospitals are closed, health personnel are absent and community health workers are close to us so we will seek help from them first”* (FGD with community members, Muyuka, SW).

Respondents in the SW region also reported that CHWs were willing to visit them in hard-to-reach locations and were available whenever they needed them. One respondent in Ekondo-Titi reported a CHW visiting her late at night, even when it was dangerous.

*“They know our situation, especially running into the bush. Some will go into the bushes to attend to someone who is sick”* (FGD with community members, Muyuka, SW).

In Muyuka, CHWs also reported that people come from neighbouring villages to use their services and said when they are not available, they leave their phone number so they can be contacted.

Although respondents said they value the proximity of CHW services, the distribution of CHWs is uneven. Both community members and CHWs in both regions reported there were not enough CHWs to cover the entire population. Community members said CHWs are part-time and are not well paid, so they are often not available as they are carrying out income-generating tasks to provide for their families. CHWs and community leaders suggested the reduced availability was due to an influx of IDPs.

“*CHWs are not always available like hospitals, they have other activities. They are part-time workers because they are not paid fully but given incentives. They are never available 24/7*” (FGD with community members, Tombel, SW).

Other respondents said that CHWs are not given accommodation to stay in the community and do not have their own transport, so often when they are called for help, they have no way to get to the person who is sick. Community members also mentioned that a lack of transport affects how well CHWs ca perform their role, as it affects how quickly they can travel time between villages and transport severely ill patients to hospital.

“*The CHWs are not given accommodation, facilities for transportation, even means of communication. So, if you were having a means of transportation if I give you a call now that I’m sick you will not be able to transport yourself. That has led to many deaths because of the above*” (IDI with a community leader, Muyuka, SW).

CHWs said to improve their availability they require better clothing and tools to work in the rainy season and at night. CHWs felt providing them with phones to record patient data instead of using paper records would also facilitate data transfer to quarter heads.

#### Affordability

Several respondents reported affordability as a key reason they visited CHWs for health care. Respondents said that first line treatment from CHWs is either free-of-charge or there are flexible payment options. Respondents said this was not the case at hospitals.

*“We will consider asking them help because their services are free-of-charge, even up to the point that we are given free drugs”* (FGD with community members, Tombel, SW).

However, other respondents mentioned the cost of treatment was a barrier to using CHWs. Participants from both the SW and Littoral regions said they are frequently ill but are unable to pay for treatment. The inability to pay for malaria treatment and purchasing roadside drugs without prescription were the most common barrier raised in all health areas in the SW and Littoral regions.

“*If you go to houses today, as mine, my son, the last child is affected by malaria. If not that the health centre is close to me, and I had some money, we would have lost the child.*” (FGD with community members, Ekondo-Titi, SW).

In Ekondo-Titi, Kumba, Muyuka, Nkongsamba and Tombel, community members reported that CHWs were selling drugs that should be free and this made their treatment unaffordable. Community leaders in Muyuka and Tombel also mentioned that affordability was a problem for their communities. One community leader reported that where health facilities are operational, there is often a mismatch between the referral offered by CHWs and the health staff in these facilities and community members complain of having to pay out-of-pocket when directed to the health centres after a consultation with the CHWs in their localities. Multiple CHWs and community members in different locations reported that in the event of a hospital referral, they anticipate treatment to be expensive, and acknowledge how some people would prefer to buy treatment from a street vendor or use herbal remedies instead.

*“Because of poverty, the people in my community are unable to follow-up for proper treatment. They just carry out casual treat through the use of traditional leaves and herbs. Those who have the means do go to hospitals while some with little cash buy from local pharmacies.”* (IDI with a community leader, Tombel, SW)

Interviewed respondents also stated the sale of malaria drugs by CHWs presents a huge challenge.

“*From the report we had from other areas in a project in Ndian we got that most of the CHWs sell out drugs which are meant to be free and since the community is already calling them doctors, they don’t tend to listen to the hierarchy*” (IDI with a member of a community-based organisation, Kumba, SW).

Respondents advised that stakeholders supporting community health should work closely with the CHWs, health management teams and health facility committees to ensure that people do not pay for antimalarials in the public health sector. CHWs in Kumba said that treatment should be free for everyone and not solely children under five.

#### Perceived quality of care

The perceived quality of care received from CHWs varied across health areas. In Muyuka, respondents reported using CHW services as they are well-trained and there was a lack of doctors or other health staff.

*“I don’t see any problem because they have been trained and since there are limited number of doctors that’s why community health workers have been sent to work.”* (FGD with community members, Muyuka, SW)

In Nkongsamba, community members said that despite initial scepticism when the CHW initiative was first introduced, they were now very satisfied with the quality of care received.

*“At the beginning it was not easy for people in the community to accept that one of theirs has just been transformed into a CHW and is proposing to treat them. But today many people are very happy with the initiative and frequent CHWs when the have health issues”* (FGD with community members, Nkongsamba, Littoral)

However, in other health areas, respondents reported doubts about the competence of CHWs. The majority of respondents from Ekondo-Titi and Tombel said they prefer to seek treatment for malaria by going directly to the hospital as they perceive the quality of care is better.

“*For individuals who doubts their capacity to handle these malaria management, they can prefer to go to the hospital rather than going to CHWs*” (IDI with a community leader, Tombel, SW).

Similarly, in Muyuka and Tombel, respondents reported having a lack of confidence in the ability of CHWs to diagnose and treat malaria due to their training. In Muyuka, respondents believed that as CHWs received less training than nurses they lacked necessary competencies.

*“No, we don’t have much trust because we don’t know their educational backings e.g., nursing, or medical school. I understand that they are trained but it’s not enough.”* (IDI with a community leader, Muyuka, SW).

A city council staff member reported that supportive supervision had been used in their area to improve the quality of care provided by CHWs and that this had been helpful to motivate CHWs to provide high-quality care.

*‘‘The best person who can ascertain the functionality of the CHWs is the chief of centre (CoC) because she receives direct monthly reports from the CHWs to key into the system and evaluates their activities, rendering them to receive some motivation at the end of the month and which is not usually regular due to some challenges’’* (IDI with a city council staff member, Tombel, SW).

#### Resourcing

Several community members reported that they did not visit CHWs as they lacked the tools to diagnose and treat malaria. In Kumba, community members said they do not know where to go for treatment, that there is no one who can test for malaria, and available services are only from non-government organizations who are only contacted when someone is critically ill.

CHWs confirmed this reporting that they have difficulty diagnosing and treating malaria because they do not have steady supply of diagnostic tests and drugs.

“*There is regular stock out of drugs which is a problem and when you refer the patients they will complain of financial difficulty. Like now there has been a stock out of drugs for 6 months now*” (FGD with CHWs, Muyuka, SW)

Community members, community leaders and a regional government staff member in Tombel reported that CHWs often do not have the drugs to treat malaria and instead refer the patient to the hospital for treatment.

*“It is challenging for CHWs to have drugs. Their drugs are not regularly available first of all at the level of the drug fund. Also, at the level of their health areas, they have challenges with availability of drugs*” (IDI with a regional government staff member, Tombel, SW).

Community leaders in Ekondo-Titi and Manjo also said the scarcity of drugs was a major concern for them, and stockouts of subsidised treatments had led to a rise in prices.

*“At times, the subsidised drugs with the CHWs are not available and when we come to the quarter are bound to buy at exorbitant prices”* (IDI with a community leader, Manjo, Littoral).

Community members in Nkongsamba said they were discouraged from using CHW services as the medicines they received from them were close to their expiry date. In Kumba, some community members also said they do not trust the medicines they are given by CHWs.

#### Awareness

In some areas, respondents did not use CHWs due to a lack of awareness of their existence or the services they provide.

“*How do we even interact with someone whose existence is not known? Many people in the community are not even aware that within them there are CHWs, and this is partly because a good number of them do not make efforts to come closer to the members of their communities with the information they have”* (FGD with community members, Manjo, Littoral)

CHWs in Manjo also reported that only around half of community members use their services as there are no clear benefits to visiting a CHW over a health facility.

#### Trust

Some respondents said they did not use CHW services as they did not trust them. Distrust was related to perception of CHWs’ motivation, their links to government, associations with COVID-19 vaccine promotion, local relationships and trust in their training and competency. In Kumba, some respondents reported not trusting CHWs due to a perception that they were government agents. In Muyuka, CHWs said community members are worried they will be given the COVID-19 vaccine if they use their services. Other barriers to trust in CHWs were feeling uncomfortable discussing health issues with someone within their community and perceiving that CHWs were giving some members of the community preferential treatment.

*“This crisis has caused a lot of issues and so much sensitization is needed as many are sceptical now even of the CHWs thinking they are government agents.”* (FGD with community members in Kumba, SW)

##### Language

In two areas of the Littoral region, language was reported to be a barrier for community members to use of CHWs. In Mbanga, IDPs who do not speak French are more likely to seek treatment elsewhere as CHWs have limited or no English. In N’lohe and Kolla, some participants said that IDPs would visit a hospital instead of using CHWs due to the language barrier, however IDPs are now becoming more comfortable with using CHW services.

*“In my zone language is a great barrier since a good number of IDPs are English speaking and hardly come to me when they have health problems and at times some prefer to leave the community to go to Mbingo in Tombel where the feel comfortable but we are making efforts to reach them especially those who speak French”* (FGD with CHWs in N’lohe and Kolla, Littoral)

### Perceptions of community engagement approaches

#### Existing community engagement approaches

When asked about existing community engagement practices many CHWs said there were no community engagement activities currently being implemented. However, a few CHWs described door-to-door campaigns to raise awareness of malaria and environmental management practices to reduce mosquito breeding sites. There were a variety of delivery mechanisms for these awareness raising activities. In Muyuka, community members reported that health communication activities are normally delivered by the town crier or CHWs, who gather community members together to provide health education and to carry out community clean up campaigns (“clear bushes”, “dig gutters”, “provide better drainage systems”). Community members in Muyuka also described CHWs conducting door-to-door household education sessions on how to use insecticide-treated nets. Similarly, in Kumba, CHWs reported carrying out awareness raising activities using a community dialogue approach. In Ekondo-Titi community leaders reported that ward leaders are normally responsible for gathering the community together for health talks. However, many participants reported that, since the start of the conflict, some of these community engagement activities had ceased.

*“Before the crisis we use to have some health programs and committee who will go round advising the community on preventive ways of malaria”* (FGD with community members, Muyuka, SW)

In comparison, in Nkongsamba, community members reported existing community dialogues, where community members meet once a week to discuss “issues” including those concerning health. Other community members reported that health education group talks had also previously been implemented.

*“In our community already, we mostly meet on Fridays to evaluate the problems of our community especially concerning health and together look for possible solutions”* (FGD with community members, Nkongsamba, Littoral)

In Kumba, community members reported that the health centre had previously implemented something like a scorecard approach, but this had not been used at village level; no other use of community scorecards was not reported in any of the other FGDs.

Participants in Ekondo-Titi, Kumba, Muyuka and Tombel mentioned village health committees either existed or had been previously used in many locations but were disrupted by the conflict. Despite community members perceiving village health committees as a positive approach that could help to solve issues relevant to the community, some participants highlighted that it could be challenging to implement due to linguistic barriers and people would only attend meetings if incentives such as food and drinks were provided.

#### Perceptions of community engagement approaches

In general, participants of the FGDs responded positively to the proposal of community engagement approaches. Community members and CHWs felt community-centred approaches would help to engage everyone and overcome current challenges with community participation. CHWs, community members and regional-level stakeholders mentioned several factors that could limit the success of community-based approaches in general, these included: a lack of participation from the community if incentives could not be provided, or if travel was affected by adverse weather or security concerns. There was also concern raised over potential disagreements in meetings, difficulties with power dynamics, a lack of a meeting venue, and language barriers, particularly concerning the preferred use of non-local languages. A member of a community-based organisation also said that said engagement with the community leader prior to the introduction of new methods was important for an approach to be successful.

“*If before implementation you meet with the community leaders, religious leader etc. who have influence on the community then I think all will go on well*” (IDI with a member of a community-based organisation, Kumba, SW).

#### Community dialogue

In Kumba, Muyuka, Nkongsamba, N’lohe and Kolla and Tombel, community members and CHWs responded positively to the community dialogue approach. Respondents said this approach was good at addressing the entire community, and that it had been helpful to learn how to prevent diseases. However, community members also reported that the provision of treatment would also be needed, as education alone will not help anyone who contracts malaria.

*“Door-to-door sensitisation being done by us is always time consuming and often we don’t get to reach everyone. But when the people are called to gather in a place it will be very easy”* (FGD with CHWs, Kumba, SW)

The community-based organisation said all the community engagement approaches would be beneficial and gave a preference for the community dialogue approach, as the community would be more open to giving their opinion. Similarly, in Kumba, Nkongsamba, and N’lohe and Kolla, community members and CHWs said they preferred this approach as they felt it would give everyone the opportunity to be involved and was more likely to reflect the expectations of the community when compared with village health committees. Community leaders in Manjo, N’lohe and Tombel said they also preferred this approach.

*“I think the community dialogue will give a chance for us at the base to come up with proposals that will guide decisions that will be taken concerning us at the top level and will provide solutions that reflect our expectations”* (FGD with community members, Nkongsamba, Littoral)

Regional government staff in Manjo and CHWs in Manjo and Mbanga felt this approach would be most successful if it were combined with the village health committee approach.

Despite this preference for community dialogue approaches for community engagement in malaria prevention and control, respondents had mixed views how effective this approach would be. Some respondents reported this approach would help the community to feel invested in the solutions and encourage participation but felt not everyone would comply with the decisions taken. Similarly, some community members and the community leader in Ekondo-Titi felt the community dialogue approach may be challenging to implement in their area due to a lack of participation from the community.

#### Scorecard

Respondents in several health areas responded positively to the scorecard approach and felt it would consider everyone’s views, help to identify individual weaknesses, and capture the thoughts of community members who do not like to speak up in other forums. In addition, community members in Muyuka felt the scorecard approach would help to improve the quality of services delivered by CHWs, and CHWs in the same area felt this would be an effective way for the community to prioritise problems and encourage its members to work towards solutions. Participants in Kumba and N’lohe and Kolla felt the community scorecard approach would work well if implemented together with the community dialogue approach.

*“It is an effective way in that you don’t have any barrier and you can be objective because your decision will not be influenced by anybody”* (FGD with community members, Muyuka, SW)

In comparison, some community members in Ekondo-Titi, Kumba and Tombel felt there may be challenges with this approach. Some were concerned that this approach may not be inclusive for members of the community who are visually impaired or where there are language or literacy barriers.

*“We have French speaking participants here and those who have problems with their eyes, so they won’t be able to read”* (FGD with community members, Tombel, SW)

#### Village health committees

Respondents in Ekondo-Titi, Kumba, Manjo, Muyuka, N’lohe and Kolla and Tombel responded positively to the village health committee approach. Community members in Manjo felt this approach would be useful for awareness raising, organising environmental management activities, and discussing sanctions for those who do not follow environmental management practices. In Ekondo-Titi, Kumba and N’lohe and Kolla, respondents felt village health committees would provide a forum for everyone to participate, mobilise resources and enhance adherence to the decisions made.

*“The village health committee can also help us organise clean-up campaigns with the collaboration of the youths. It will also establish sanctions for people who do not respect hygiene measures”* (FGD with community members, Manjo, Littoral)

Community members in Ekondo-Titi, Kumba, Muyuka and Tombel raised potential challenges they foresaw with implementing the village health committee approach. These challenges included the need for support to set up these committees, the sustainability of the approach if committee members were unpaid, other organisations taking advantage of the training provided and employing its members, the need to include health professionals in the committee, the importance of gender balance among the committee members, and the potential emergence of tensions between communities and CHWs.

## Discussion

This study confirmed that since the beginning of the conflict in Cameroon, access to preventive and curative health services has become increasingly limited. The attacks on health centres have reduced the number of functioning facilities, and restrictions on movement have limited travel to those that remain. This is consistent with relevant UNOCHA reports [4, 5, 7].

CHWs have been shown to be an effective way to maintain access to health services during conflict events [14]. In Cameroon CHWs are recruited in the community by district health supervisors, heads of health facilities and community leaders selected according to an established criteria stated in the community health guidelines and community health worker terms of reference. They undertake six months of training that covers key modules including: household registration, surveillance, integrated community case management, maternal and newborn health, sexual and reproductive health, and communication, reporting and drug management. Drugs and medical supplies are provided through the Ministry of Health and implementing partners with funding from organisations such as the Global Fund to fight AIDS, tuberculosis and malaria. Drugs and medical supplies are provided through a pull system based on CHW orders and medical reports. The medicines are supplied to focal health facilities and are available for collection on delivery of monthly medical reports. Allowances are also made for emergency orders. The National Malaria Control Programme, district supervisors and implementing partners report ongoing challenges with this system including supply bottlenecks, limited supervision of CHW drug management and funding constraints. This research confirmed that the provision of CHWs, as part of the Cameroon Ministry of Health’s Community Health Strategy, has improved access to health services for communities living in the conflict-affected SW region, and has provided an additional avenue for receiving care for communities in the Littoral region.

Despite improvements in access to CHWs, this study identified several reasons why CHWs are not fully utilised by the community, and possible improvements to ensure CHWs can provide high-quality care. There were frequent reports across the study sites that community members were unaware of CHWs and the services they provided. This resulted in caregivers unnecessarily travelling long distances for healthcare or not seeking professional treatment at all. Increasing the visibility of CHWs and the services they provide could improve treatment-seeking rates and result in a reduction in malaria cases.

In addition, in areas where community members were aware of CHWs in the area, many did not use their services due to poor availability, lack of affordable services and a perception that they were not able to provide high-quality care, either due to a perceived lack of training or due to stockouts of health commodities.

The study found a lack of availability of CHWs was due to CHWs living in neighbouring communities to avoid conflict zones, and sometimes only able to travel to some communities when the roads are safe and passable. In addition, in some areas, CHWs must balance their work as a CHW with other income-generating activities to provide for their own family; this could also explain why some CHWs were reported to be selling treatments instead of providing them for free. Strategies to improve availability could include increasing the number of CHWs in each community and providing CHWs with an improved salary, transport allowance and accommodation so they can stay in the community or quickly travel when needed. The provision of transport would also allow CHWs to manage simple referrals themselves. Studies have shown that the performance of CHWs is higher where incentives—financial or not—are provided and supervision and training is regular [15–17]. Providing a salary may also decrease the likelihood of treatments being sold for a profit and therefore increase the affordability of services for community members.

Community members also reported a lack of availability of treatments, CHWs were not sufficiently equipped to treat malaria. CHWs were also particularly concerned about stockouts, and community leaders also confirmed that there are times when CHWs do not have the tools they need to perform their role. The lack of availability of healthcare workers, and associated problems with the supply of medicines, has also been reported as a barrier to uptake of CHWs in other countries, such as Kenya [18]. It has also been reported that when people do not receive the appropriate drugs due to frequent stockouts, they develop negative perceptions of care providers and have limited trust in the health system [19]. More effort is needed to ensure CHWs are fully equipped to provide high-quality services.

Regarding acceptability of treatment services, most community members reported they were satisfied with the quality of the services provided by the CHWs. The main reasons given were availability and effectiveness of the treatment they provided when they had stock. However, a minority of community members in the study setting appeared to have strong negative perceptions of CHWs’ ability to provide good quality care, as they had not received formal medical training. This was consistent with other studies conducted in sub-Saharan Africa which found perceived training to be a factor affecting use of CHWs [20–22]. To build trust in CHW services community members should be made aware of the training CHWs have received.

Affordability is a major barrier to malaria treatment and healthcare throughout sub-Saharan Africa, and especially in conflict-affected communities where households have lost their primary source of income due to conflict [23, 24]. The study found that although the Cameroon Government provides free treatment for children under five and pregnant women with uncomplicated malaria, respondents still reported cost-related barriers to using CHWs services. Ensuring services are free at the point-of-care and properly administered will help to reduce the amount of people seeking alternative healthcare options.

This study found that community-based approaches were perceived to be appropriate methods for reducing some of these challenges, including raising awareness of the presence of CHWs and their training. The preferred method for community engagement was the community dialogue approach. However, the majority of participants responded positively to all three proposed approaches. If implemented, the scorecard and village health committee approaches would need to be adapted to ensure they are inclusive and representative of all members of the community.

These findings align with several other studies which have demonstrated the successful use of the community dialogue approach for integrated community case management of malaria, pneumonia and diarrhoea in Mozambique, Uganda and Zambia [25, 26]. This approach has been effective at filling health information gaps and helping communities make collective decisions to improved health practices and has demonstrated feasibility in low resource settings [27]. In addition, a study exploring community dialogues for child survival in Uganda highlighted the importance of participatory action, follow up, and post-meeting monitoring as a critical component to the community dialogue process [28]. To date, community engagement approaches have focused more on encouraging prevention activities and supporting CHWs to provide health education to the community, but not for providing a platform for communities to advocate for better supplies and support for CHWs. This approach has been shown to increase community uptake of integrated community case management services and could be a useful tool to increase visibility of CHW services in communities where a lack of awareness was reported [26].

The findings from this study have led to a recommendation to review support given to CHWs. A study on interventions for community health service access in Mozambique and Uganda determined that interventions should promote CHWs as members of a collective, by highlighting a sense of shared experience, focus on alignment between worker and programme goals, and emphasise the actions that lead to good performance [29]. By increasing supportive supervision CHWs have also be shown to feel more connected to the health system and their community, are motivated by status and community standing, and want to be provided with the necessary tools to perform [29].

Rowe et al*.* conducted a large systematic review of health care provider performance in low- and middle-income countries concluding that a multiple layer approach that included training, supervision, adequate resources, links to the health system peer support and community support would improve performance [30]. The study reviewed CHW research and found results were largely inconclusive but recommended that training lay health workers seemed to have a small effect size, and combining training with community support might be more effective.

A recent study evaluated the CHW supervision system developed by the President's Malaria Initiative, the outreach training and supportive supervision approach [31]. The study cited well known barriers to CHW performance of human resources and supply chain management, challenges with designing, implementing, and sustaining supportive supervision approaches, an absence of feedback after visits, irregular visits, poor continuity between visits, top-down planning, and a failure to adhere to supervision plans because of inadequate resources for implementation. This study reported their participants valued supervision checklist, quality supervisor supervisee interactions and cited studies that found onsite supervision, positive experience of onsite mentoring and coaching visits and peer support are highly valued by CHWs.

The use of the community scorecard approach and village health committee could also be implemented in conjunction with the community dialogue approach to overcome challenges such as trust in CHWs and improve the quality of services they provide by monitoring training and adherence to guidance. The study by Martin et al*.* found the community dialogue approach was effective in communities where there are low levels of literacy which was reported as a challenge to using the scorecard approach in some of the communities studied [27].

### Strengths and limitations

The strength of this work is that it provides information for a previously understudied area. However, conducting research in a conflict-affected area is not without challenges and several limitations should be considered with regards to the interpretation of the findings. In some of the FGD transcripts with community members, the qualitative data analysts perceived that some of the participants could have previously been a CHW, which may have introduced desirability bias and affected responses. During transcript analysis, it was noted that questions asked in FGDs with CHWs were not identical across different regions. Other limitations include positionality differences between the study participants and the research team: gender, culture and language could have impacted the experiences described in this study.

## Conclusion

The ongoing conflict in Cameroon is negatively affecting the population’s access to healthcare, including services to prevent and treat malaria. The findings of this study show that although the Ministry of Health has developed a strong Community Health Strategy and deployed CHWs to ensure continuity of health access, more must be done to remove barriers that discourage the community from using this source of care. The study found that the community dialogue approach could be an effective tool to remove some of the barriers including raising awareness of CHWs and the training they receive and building trust between community members and CHWs. However, implementing a community dialogue approach alone will not be sufficient to remove all the barriers identified. Communities and stakeholders expressed that CHW services could be improved by focusing on supervision, resourcing and referral support, to improve trust in services. Further research is needed to determine the cause of these issues and develop tailored solutions.

The results of this study have been used to design a project using three key interventions to enhance the quality of malaria prevention and control services: an adapted community dialogue approach (the Community Health Participatory Approach), improved support for CHWs, and vouchers to cover the cost of transport to and treatment from a CHW or health facility. This project is currently being implemented in 80 communities in SW and Littoral regions. As countries across the world move closer to malaria elimination, more must be done to support conflict-affected areas where malaria case management is more challenging, to ensure they are not left behind.

### Supplementary Information

**Additional file 1. **Topic guide for focus group discussions with male and female community members and community health workers to assess health-seeking behaviour and knowledge surrounding malaria prevention and control and preference for community engagement approaches in conflict-affected communities of Cameroon.

**Additional file 2. **Topic guide for in-depth interviews with key local and regional stakeholders to assess health-seeking behaviour and knowledge surrounding malaria prevention and control and preference for community engagement approaches in conflict-affected communities of Cameroon.

## Data Availability

The datasets used and/or analysed during the current study are available from the corresponding author on reasonable request.

## Abbreviations

CHW: Community health workers
FGD: Focus group discussion
IDI: In-depth interviews
IDP: Internally displaced person
NW: Northwest
SW: Southwest
UNOCHA: The United Nations Office for the Coordination of Humanitarian Affairs
