# Supplementary material for: Exploring existing malaria services and the feasibility of implementing community engagement approaches amongst conflict-affected communities in Cameroon: a qualitative study
Source: Malar J. 2024 May 20;23:155. doi: 10.1186/s12936-024-04934-x (PMC11107007; doi:10.1186/s12936-024-04934-x)
Supplement: Supplementary file 1 — Additional file 1. Topic guide for focus group discussions with male and female community members and community health workers to assess health-seeking behaviour and knowledge surrounding malaria prevention and control and preference for community engagement approaches in conflict-affected communities of Cameroon. [file 12936_2024_4934_MOESM1_ESM.docx]

## **Topic guide for focus group discussions**

### Community members

**A. Malaria Services Provided by Community Health Workers**

1. We are interested in understanding more about malaria in your community and the care you receive.
   1. How serious is malaria a problem in your community?
   2. What makes you think it is serious?
   3. Why do you think it is not serious?
   4. What do you do if you suspect that you or your child has malaria? How could seeking the service of your CHW be helpful?
   5. What would be the reason for you not wanting to seek their help?
2. How easy is it for you and other community members to access effective treatment when you feel like you have malaria?
   1. What makes it easy?
   2. What might be the reason some people find it difficult to get this care?
   3. How has the conflict and displacement affected your ability to access malaria treatment?
3. The intervention we want to test is to strengthen appropriate health-seeking behaviour in your community, in particular malaria care given by Community Health Workers.
   1. How often do you generally interact with Community Health Workers?
   2. If yes, where do you usually meet with them?
   3. If never, why not?
   4. Can you describe how you usually interact with Community Health Workers?
   5. What kind of tasks and responsibilities do the Community Health Workers usually have?
4. How well are the Community Health Workers able to treat malaria in their communities?
   1. Could you elaborate what makes you think so?
   2. In your opinion, what are the challenges when Community Health Workers treat malaria in their own communities?
   3. How could the problems you mentioned be addressed?
   4. What could be done differently to improve this?

**B. Community Engagement**

1. Community Health Workers in this area were trained through the Global Fund to provide appropriate health care for malaria for all community members. What are the responsibilities of Community Health Workers when it comes to malaria care?
   1. What is your understanding of the term ‘community engagement’?
   2. How is it currently done in your own community?

Let’s look at the ways to do community engagement in more detail. We have prepared a fact sheet that provides some information about some different approaches.

[Hand out a community engagement fact sheet. Read out the information, one approach at a time, before you discuss that approach. Give people time to look at the steps involved and provide an opportunity to ask clarification questions.]

Community Dialogues

1. Have you ever seen people in your community doing something similar to discuss health related issues, especially about malaria?
   1. If yes, what were the things which people did which were similar?
   2. Do you think that was an effective way for community members to discuss health issues? Why/why not?
   3. From your understanding of the Community Dialogue approach, do you think it would be a good way to enable the community to make decisions about their own health?
   4. If yes, why do you think that?
   5. If not, why do you think not? Is there any way the approach could be easily changed to make it more effective?

Community Scorecards

1. Have you ever seen people in your community doing something similar to discuss health related issues, especially about malaria?
   1. If yes, what were the things which people did which were similar?
   2. Do you think that was an effective way for community members to discuss health issues?
   3. From your understanding of the Community Scorecard approach, do you think it would be a good way to enable the community to make decisions about their own health?
   4. If yes, why do you think that?
   5. If not, why do you think not? Is there any way the approach could be easily changed to make it more effective?

Village Health Committees

1. Have you ever seen people in your community doing something similar to discuss health related issues, especially about malaria? This may be through Village Health Committees, or other community groups such as “men’s clubs” or “mothers’ meetings”.
   1. If yes, what were the things which people did which were similar?
   2. Do you think that was an effective way for community members to discuss health issues?
   3. From your understanding of the Village Health Committee approach, do you think it would be a good way to enable the community to make decisions about their own health?
   4. If yes, why do you think that?
   5. If not, why do you think not? Is there any way the approach could be easily changed to make it more effective?
   6. In your community, do community-based organisations help the community to access malaria treatment? If yes, how helpful do you think this is?

**C. Overall perception of the interventions**

1. What do you generally think of the idea of introducing a Community Engagement approach like one of the three on the fact sheets we looked at together? Please note that these are just suggestions, and we would like to also hear your thoughts and suggestions.
   1. What kind of challenges do you foresee?
   2. Do you think there are any challenges with regard to gender? Would women and men face different challenges in interacting with the community engagement approaches?
   3. Do you think there is already something which the community do which would be more suitable for increasing awareness around care seeking for malaria?
   4. If yes, please describe how it works?
   5. Do you think that approach allows all community members to engage, regardless of sex, disability, or other socioeconomic factors?
2. Is there anything else you would like to share with the research team?

### Community health workers

**A. Malaria Services Provided by Community Health Workers**

1. We are interested in understanding more about malaria in your community and the care you provide.
   1. To what extent is malaria a problem in your community? Why do you say that?
   2. What kinds of activities do you offer the community? [Probe on types of outreach, e.g., home visits, community education. Probe on kinds of info and services related to malaria]
   3. Who trained and supports you to offer such information and services?
   4. Could you explain what is your relation with the nearest health facility? [Probe on supervision support, materials supply, making referrals, organizing outreach activities with the health facility.]
   5. Who do you reach most often in the community with info and services? [Probe on target groups - women, men, youth, couples] Who are the target priority groups? Why?
2. The intervention we want to test is to strengthen appropriate health-seeking behaviour in your community, in particular going to Community Health Workers for malaria care.
   1. How would you describe the main roles and responsibilities of Community Health Workers?
   2. Do people in your communities use the services of Community Health Workers?
   3. If not, why not?
   4. If yes, what do you believe are people’s opinions of the services they receive? Why do you say that?
3. Do you think the Community Health Workers are able to effectively treat malaria in their communities?
   1. If yes, why do you think so?
   2. If no, why not?
   3. In your opinion, are there any problems with tasking Community Health Workers to treat malaria in their own communities?
   4. How could the problems you mentioned be addressed? What alternatives or improvements would you suggest?

**B. Community Engagement**

1. We are interested to learn more about how the IDP, and host communities discuss and make decisions related to their health, in particular malaria.
   1. What is your understanding of the term ‘community engagement’?
   2. How is it currently done in the communities where you provide malaria care?
   3. Do you engage in any community engagement activities currently? How?

Let’s look at the ways to do community engagement. We have prepared a fact sheet that provides some information about some different approaches.

[Hand out a community engagement fact sheet. Read out the information, one approach at a time, before you discuss that approach. Give people time to look at the steps involved and provide an opportunity to ask clarification questions.]

Community Dialogues

1. Have you ever seen people in your community doing something similar to discuss health related issues, especially about malaria?
   1. If yes, what were the things which people did which were similar?
   2. Do you think that was an effective way for community members to discuss health issues? Why/why not?
   3. From your understanding of the Community Dialogue approach, do you think it would be a good way to enable the community to make decisions about their own health?
   4. If yes, why do you think that?
   5. If not, why do you think not? Is there any way the approach could be easily changed to make it more effective?

Community Scorecards

1. Have you ever seen people in your community doing something similar to discuss health related issues, especially about malaria? This may be through Village Health Committees, or other community groups such as “men’s clubs” or “mothers’ meetings”.
   1. If yes, what were the things which people did which were similar?
   2. Do you think that was an effective way for community members to discuss health issues?
   3. From your understanding of the Community Scorecard approach, do you think it would be a good way to enable the community to make decisions about their own health?
   4. If yes, why do you think that?
   5. If not, why do you think not? Is there any way the approach could be easily changed to make it more effective?

Village Health Committees

1. Have you ever seen people in your community doing something similar to discuss health related issues, especially about malaria?
   1. If yes, what were the things which people did which were similar?
   2. Do you think that was an effective way for community members to discuss health issues?
   3. From your understanding of the Village Health Committee approach, do you think it would be a good way to enable the community to make decisions about their own health?
   4. If yes, why do you think that?
   5. If not, why do you think not? Is there any way the approach could be easily changed to make it more effective?
   6. In your community, do community-based organisations help the community to access malaria treatment? If yes, how helpful do you think this is?

**C. Overall perception of the interventions**

1. What do you generally think of the idea of introducing a Community Engagement approach like one of the three on the fact sheets we looked at together? Please note that these are just suggestions, and we would like to also hear your thoughts and suggestions.
   1. What kind of challenges do you foresee?
   2. Do you think there are any challenges with regard to gender? Would women and men face different challenges in interacting with the community engagement approaches?
   3. Do you think there is already something which the community do which would be more suitable for increasing awareness around care seeking for malaria?
   4. If yes, please describe how it works?
   5. Do you think that approach allows all community members to engage, regardless of sex, disability, or other socioeconomic factors?

Is there anything else you would like to share with the research team?
