# Supplementary material for: Exploring existing malaria services and the feasibility of implementing community engagement approaches amongst conflict-affected communities in Cameroon: a qualitative study
Source: Malar J. 2024 May 20;23:155. doi: 10.1186/s12936-024-04934-x (PMC11107007; doi:10.1186/s12936-024-04934-x)
Supplement: Supplementary file 2 — Additional file 2. Topic guide for in-depth interviews with key local and regional stakeholders to assess health-seeking behaviour and knowledge surrounding malaria prevention and control and preference for community engagement approaches in conflict-affected communities of Cameroon. [file 12936_2024_4934_MOESM2_ESM.docx]

## **Topic guide for in-depth interviews**

### Community leaders

1. I would like to talk with you about how your community members take care of their health and manage malaria.
   1. How do you think people in your community can best protect themselves from getting malaria?
   2. What do you yourself advise people in your community when it comes to malaria?
   3. Where do you think they can get good care?
   4. What are the challenges your community faces in terms of malaria illnesses?
   5. Could you elaborate on what causes those challenges?
2. The government has introduced a community health worker programme in your area with support from Global Fund. The community health workers (or CHWs) have been trained to provide malaria services to all community members. This service is called community case management.
   1. How many CHWs are currently providing malaria case management services in your community?
   2. Why should people in your community use this service?
   3. What do people in your community do most often when they feel like they have malaria?
   4. Why should they seek the help of CHW when they think they have malaria?
   5. What would be the reason not to seek out CHW for this care?
3. Do you know of any challenges which make it difficult for your community members to go the CHWs when they need health care?
   1. Why do you think community members may not go to CHWs when they are ill?
   2. Do you think that your community members have much trust in the ability of CHWs to give them good care, especially for malaria?
   3. If not, why not?
   4. Does the community influence each other on whether they will go to CHWs for malaria care?
4. I would like to talk with you about a number of different approaches which can be used to get community members to discuss health issues such as malaria, and to make decisions together as a community.

[Share fact sheet on Community Dialogues and give time for them to read it; if preferred read out the text to them] Have you heard of this approach before and if yes, can you tell me when and how?

1. Have you ever applied the Community Dialogue approach for yourself, or anything you think was very similar?
2. If yes, please can you describe the experience and what did you think was good, and bad about it?
3. Do you think the approach would easily include all community members – even those who are normally not asked because of disability or something else?

[Share fact sheet on Community Scorecards and give time for them to read it; if preferred read out the text to them] Have you heard of this approach before and if yes, can you tell me when and how?

- 1. Have you ever applied the Community Scorecard approach for yourself, or anything you think was very similar?
  2. If yes, please can you describe the experience and what did you think was good, and bad about it?
  3. Do you think the approach would easily include all community members – even those who are normally not asked, possibly because of disability or something else?

[Share fact sheet on Village Health Committees and give time for them to read it; if preferred read out the text to them] Have you heard of this approach before and if yes, can you tell me when and how?

- 1. Have you ever applied the Village Health Committees approach for yourself, or anything that you think was very similar?
  2. If yes, please can you describe the experience and what did you think was good, and bad about it?
  3. Do you think the approach would easily include all community members – even those who are normally not asked, possibly because of disability or something else?

1. What do you think is the best way to support your community, and other communities, to discuss together to make decisions related to improving their own health?
   1. Do you think that any of the three approaches I showed you might be good to enable your community to do this?
   2. Are you able to name one approach as being more likely to work than the others? Or would you prefer to name another approach for engaging the community on health-related issues for them to take their own decisions?
   3. Why do you feel that way?
2. Is there anything else you would like to add before we finish the interview?

### Community-based organisation

1. I would like to talk with you about how the members of the community where you work take care of their health and manage malaria.
   1. How do you think the community members can best prevent themselves from getting malaria, and if they do, how should they get good care?
   2. In these communities, do you advise community members about their health, especially malaria?
   3. What do you advise them on?
   4. Are there any challenges in the community in terms of malaria illnesses?
   5. What are the reasons for those challenges?
   6. Does your organisation provide any additional support for the community related to malaria care? If yes, please can you describe this support? How well is it working?
2. The government has introduced a community health worker programme in your area with support from Global Fund. The community health workers (or CHWs) have been trained to provide malaria services to all community members. This service is called community case management.
   1. Do you think it makes sense for the community members to use this service?
   2. Why do you feel that way?
   3. Do you know how many CHWs are currently providing malaria case management services in your community?
   4. Do you know if your community members go to CHWs when they feel like they have malaria?
3. Do you know of any challenges which make it difficult for the community members to go the CHWs when they need health care?
   1. Why do you think community members may not go to CHWs when they are ill?
   2. Do you think that your community members have much trust in the ability of CHWs to give them good care, especially for malaria?
   3. If not, why not?
   4. Does the community influence each other on whether they will go to CHWs for malaria care?
4. I would like to talk with you about a number of different approaches which can be used to get community members to discuss health issues such as malaria, and to make decisions together as a community.

[Share fact sheet on Community Dialogues and give time for them to read it; if preferred read out the text to them] Have you heard of this approach before and if yes, can you tell me when and how?

- 1. Have you ever applied the Community Dialogue approach for yourself, or anything you think was very similar?
  2. If yes, please can you describe the experience and what did you think was good, and bad about it?
  3. Do you think the approach would easily include all community members – even those who are normally not asked because of disability or something else?

[Share fact sheet on Community Scorecards and give time for them to read it; if preferred read out the text to them] Have you heard of this approach before and if yes, can you tell me when and how?

- 1. Have you ever applied the Community Scorecard approach for yourself, or anything you think was very similar?
  2. If yes, please can you describe the experience and what did you think was good, and bad about it?
  3. Do you think the approach would easily include all community members – even those who are normally not asked, possibly because of disability or something else?

[Share fact sheet on Village Health Committees and give time for them to read it; if preferred read out the text to them] Have you heard of this approach before and if yes, can you tell me when and how?

- 1. Have you ever applied the Village Health Committees approach for yourself, or anything that you think was very similar?
  2. If yes, please can you describe the experience and what did you think was good, and bad about it?
  3. Do you think the approach would easily include all community members – even those who are normally not asked, possibly because of disability or something else?

1. What do you think is the best way to support your community, and other communities, to discuss together to make decisions related to improving their own health?
   1. Do you think that any of the three approaches I showed you might be good to enable your community to do this?
   2. Are you able to name one approach as being more likely to work than the others? Or would you prefer to name another approach for engaging the community on health-related issues for them to take their own decisions?
   3. Why do you feel that way?
2. Is there anything else you would like to add before we finish the interview?

### City Council staff

1. In your own words, can you describe the malaria case management service provided by community health workers, which is supported by Global Fund in your city and surrounding area?
   1. Do you know how the community health workers were trained?
   2. Do you know how many there are in your region and if they are functional?
   3. Do you know if the community health workers receive any supervision? If yes, can you describe how this is done?
   4. Do you know whether the community health workers have an adequate supply of RDTs and drugs?
   5. Do you receive data on the programme on a regular basis? If yes, can you tell me the data received and the frequency of reporting?
2. Do you know, in the conflict-affected areas of your city and area, how the populations (internally displaced and host) living there can prevent, and access care for, malaria? If yes, please describe what you know in your own words.
   1. Do you have any information on the levels of access to mosquito nets and their usage among these populations?
   2. Do you consider that these populations have a good understanding of appropriate actions to prevent and to successfully treat malaria?
   3. Do you know where they go for healthcare when they, or their children, fall ill with suspected malaria?
   4. Can you give details of the different types of healthcare providers which may be used for malaria care by these populations?
   5. Do you consider it is easy for these populations to access effective healthcare? Can you give some examples of what may be the main barriers to access?
3. Are you familiar with any approaches which can be used to promote appropriate healthy behaviours among communities? If yes, can you describe these in your own words?
   1. Have you ever observed any such approaches applied amongst the internally displaced populations in your region? If yes, can describe in your own words and include when and where this happened.
   2. Specifically, are you familiar with the Malaria Matchbox approach? If yes, please could you describe what this is in your own words and mention whether you know if this is, or will be, implemented in Cameroon.
   3. Based on your knowledge about it, if you have any, do you think the Matchbox approach may be a suitable way to strengthen appropriate healthy behaviour for malaria in the internally displaced populations in your region?
   4. Do you know through what channels these populations normally receive information about healthcare services and how to prevent disease? If yes, please could you share those you know about.
   5. Is there any difference between the internally displaced and host populations in how they receive this type of information?
   6. Do you know how usually these communities come to make decisions related to health which affect the whole community? If yes, please could you describe these processes in your own words.
   7. In your opinion, what would be the best forms/ways of educating people in community health care?
4. Within our project, we want to select and evaluate a suitable community engagement approach which would be designed to promote collective decision making for more appropriate healthy behaviour, especially in relation to malaria. Possible examples include community dialogues, community scorecards or village/community health clubs.
   1. Are you familiar with any of these approaches and if yes, can you describe what this in your own words?
   2. How do you think this would be useful for the communities?
   3. How would this be useful for someone in your position or the regional government health office?
   4. Should other diseases be covered through this approach?
   5. Is this approach different from other health promotion activities of which you have been part or in which you have participated? How?
   6. What would need to happen to make this approach effective in the longer term?
   7. How do you think this approach might work with the Malaria Matchbox approach to improve healthy behaviours for malaria among internally displaced populations in your region?

[Repeat these questions for each approach which the interviewee mentions]

1. What other factors may have contributed to people adopting better prevention and healthcare practices?
2. Is there something else that you would like to add or share with me?

### Regional Government Staff

1. In your own words, can you describe the malaria case management service provided by community health workers, which is supported by Global Fund in your region?
   1. Do you know how the community health workers were trained?
   2. Do you know how many there are in your region and if they are functional?
   3. Do you know if the community health workers receive any supervision? If yes, can you describe how this is done?
   4. Do you know whether the community health workers have an adequate supply of RDTs and drugs?
   5. Do you receive data on the programme on a regular basis? If yes, can you tell me the data received and the frequency of reporting?
2. Do you know, in the conflict-affected areas of your region, how the populations (internally displaced and host) living there can prevent, and access care for, malaria? If yes, please describe what you know in your own words.
   1. Do you have any information on the levels of access to mosquito nets and their usage among these populations?
   2. Do you consider that these populations have a good understanding of appropriate actions to prevent and to successfully treat malaria?
   3. Do you know where they go for healthcare when they, or their children, fall ill with suspected malaria?
   4. Can you give details of the different types of healthcare providers which may be used for malaria care by these populations?
   5. Do you consider it is easy for these populations to access effective healthcare? Can you give some examples of what may be the main barriers to access?
3. Are you familiar with any approaches which can be used to promote appropriate healthy behaviours among communities? If yes, can you describe these in your own words?
   1. Have you ever observed any such approaches applied amongst the internally displaced populations in your region? If yes, can describe in your own words and include when and where this happened.
   2. Specifically, are you familiar with the Malaria Matchbox approach? If yes, please could you describe what this is in your own words and mention whether you know if this is, or will be, implemented in Cameroon.
   3. Based on your knowledge about it, if you have any, do you think the Matchbox approach may be a suitable way to strengthen appropriate healthy behaviour for malaria in the internally displaced populations in your region?
   4. Do you know through what channels these populations normally receive information about healthcare services and how to prevent disease? If yes, please could you share those you know about.
   5. Is there any difference between the internally displaced and host populations in how they receive this type of information?
   6. Do you know how usually these communities come to make decisions related to health which affect the whole community? If yes, please could you describe these processes in your own words.
   7. In your opinion, what would be the best forms/ways of educating people in community health care?
4. Within our project, we want to select and evaluate a suitable community engagement approach which would be designed to promote collective decision making for more appropriate healthy behaviour, especially in relation to malaria. Possible examples include community dialogues, community scorecards or village/community health clubs.
   1. Are you familiar with any of these approaches and if yes, can you describe what this in your own words?
   2. How do you think this would be useful for the communities?
   3. How would this be useful for someone in your position or the regional government health office?
   4. Should other diseases be covered through this approach?
   5. Is this approach different from other health promotion activities of which you have been part or in which you have participated? How?
   6. What would need to happen to make this approach effective in the longer term?
   7. How do you think this approach might work with the Malaria Matchbox approach to improve healthy behaviours for malaria among internally displaced populations in your region?

[Repeat these questions for each approach which the interviewee mentions]

1. What other factors may have contributed to people adopting better prevention and healthcare practices?
2. Is there something else that you would like to add or share with me?
